# Vehicular Sensing for Improved Urban Mobility

**DOI:** 10.3390/s24165134

**Published:** 2024-08-08

**Authors:** Constantin-Florin Caruntu, Ciprian-Romeo Comsa

**Affiliations:** 1 Department of Automatic Control and Applied Informatics, “Gheorghe Asachi” Technical University of Iasi, 700050 Iasi, Romania; 2 Department of Telecommunications and Information Technologies, “Gheorghe Asachi” Technical University of Iasi, 700506 Iasi, Romania; ccomsa@etti.tuiasi.ro

In recent years, advancements in the automotive industry have accelerated the development of connected and autonomous vehicles (CAVs). While fully autonomous vehicles still require significant progress to meet all safety and security standards, there are emerging opportunities to enhance traffic safety using environmental sensor data and connectivity with other vehicles and smart infrastructure, especially in urban settings.

Environmental sensors such as cameras, radars, and lidars provide critical information about objects and other traffic participants around vehicles. The latest CAV developments enable the sharing of this information between vehicles and infrastructure, known as collective perception. Vehicles can now create and improve their environment models (EMs) with data from their own sensors and from vehicle-to-everything (V2X) communication technologies. This allows vehicles to transmit their heading, position, and speed through cooperative awareness messages (CAMs) and enhance precision with collective perception messages (CPMs).

Urban infrastructure, equipped with various sensors, like cameras, radars, and GNSS, plays a crucial role in traffic safety. Smart infrastructure employs computer vision and artificial intelligence (AI) for object detection, classification, pose estimation, tracking, and behavior prediction, thus enhancing traffic participants’ awareness. Thus, this Special Issue explores all aspects of vehicular sensing in urban mobility, including architecture, emerging sensors, communication technologies, advanced applications, and deployment issues, such as developing smart infrastructure systems for information sharing and safety.

The rapid expansion of urban environments necessitates innovative solutions for improving mobility and reducing congestion. Vehicular sensing technologies hold significant promise in addressing these challenges by enhancing traffic management, ensuring safety, and optimizing vehicle performance. This Special Issue, titled “Vehicular Sensing for Improved Urban Mobility”, showcases ten cutting-edge studies that advance the state of the art in vehicular sensing and its applications in urban settings.

## 1. Overview of the Contributions

In their study, Fernández et al. [1] develop a dual-slope path loss model tailored for vehicular sensing applications in diverse urban and suburban environments. This model aims to improve the reliability and accuracy of vehicular communications, which are crucial for the deployment of intelligent transportation systems (ITSs) and vehicular ad hoc networks (VANETs).

Herbers, Doerzaph, and Stowe [2] explore how line-of-sight (LOS) sensors and connected vehicle technology (CVT) can mitigate and prevent crash and near-crash scenarios. Their research demonstrates the potential of these technologies to enhance vehicle safety through advanced driving assistance systems (ADASs).

Heyer-Wollenberg et al. [3] propose a cooperative method to improve the accuracy of Turn Movement Count (TMC) by incorporating contextual observations from surrounding areas. This method significantly enhances the identification of vehicle movements under challenging conditions, contributing to more accurate traffic analysis and management.

In their paper, Silva et al. [4] present a model-based approach to quantify the dependability of VANETs, particularly in urban advanced mobility (UAM) contexts. By leveraging virtual machine migration, they aim to enhance the reliability and availability of VANETs, which are essential for integrating UAM into urban infrastructures.

Lazar et al. [5] discuss a comprehensive control architecture for connected vehicle platoons, utilizing vehicle-to-everything (V2X) communication. This architecture improves road safety, traffic flow, and fuel efficiency, offering a promising solution to contemporary traffic problems.

Shopovska et al. [6] address the challenge of detecting vulnerable road users (VRUs) under varying lighting conditions. They introduce a high-dynamic-range tone mapping technique for intelligent automotive systems, enhancing the performance of imaging sensors in extreme lighting scenarios.

Achirei et al. [7] present a model predictive control framework for omnidirectional mobile robots, emphasizing the use of convolutional neural networks (CNNs) for object detection. Their approach significantly improves the navigation and operational efficiency of mobile robots in urban logistic environments.

Frej et al. [8] conducted an experimental study on the longitudinal acceleration of urban buses and coaches, analyzing vehicle motion dynamics and driver behavior. Their findings provide valuable insights for enhancing passenger comfort and safety in urban public transportation.

Park et al. [9] propose a secure mutual authentication and key agreement scheme using physically unclonable functions (PUFs) for Internet of Drones (IoD) applications. This scheme ensures robust security in UAV operations, which is crucial for urban traffic surveillance and environmental monitoring.

Zhao and Zhao [10] developed an algorithm for online stochastic error modeling of inertial sensors used in urban navigation systems. Their approach enhances the accuracy of inertial navigation during GNSS outages, which is vital for reliable vehicle positioning in dense urban areas.

## 2. Conclusions

This Editorial provides an overview of each paper’s contributions to the field of vehicular sensing and emphasizes their impact on improving urban mobility. The papers in this Special Issue highlight the diverse applications and significant advancements in vehicular sensing technologies. By addressing key challenges in urban mobility, these studies contribute to safer, more efficient, and sustainable transportation systems.

We, the Editorial Team, appreciate all the innovative research endeavors presented in this Special Issue. We extend our thanks to the authors for their diligent incorporation of feedback, critical assessment of their work, and adherence to timelines, which have enabled the successful publication of this Special Issue. The Guest Editors are pleased with the conclusive outcomes of the published papers and anticipate their utility for researchers, engineers, designers, and other professionals engaged in various aspects of advanced analytical and numerical simulation approaches, as well as experimental studies applied to vehicular sensing and urban mobility. We also express our gratitude to the reviewers for their crucial contributions and the dissemination of scientific findings. Lastly, we thank the Editorial Board of *Sensors* for their patience, support, and exceptional contributions. We hope the readers feel inspired by and can learn from the research articles in this Special Issue.
